# Supplementary material for: Transcription-dependent cohesin repositioning rewires chromatin loops in cellular senescence
Source: Nat Commun. 2020 Nov 27;11:6049. doi: 10.1038/s41467-020-19878-4 (PMC7695716; doi:10.1038/s41467-020-19878-4)
Supplement: Supplementary file 1 — Supplementary Information [file 41467_2020_19878_MOESM1_ESM.pdf]

Supplementary Fig. 1_Olan et al.

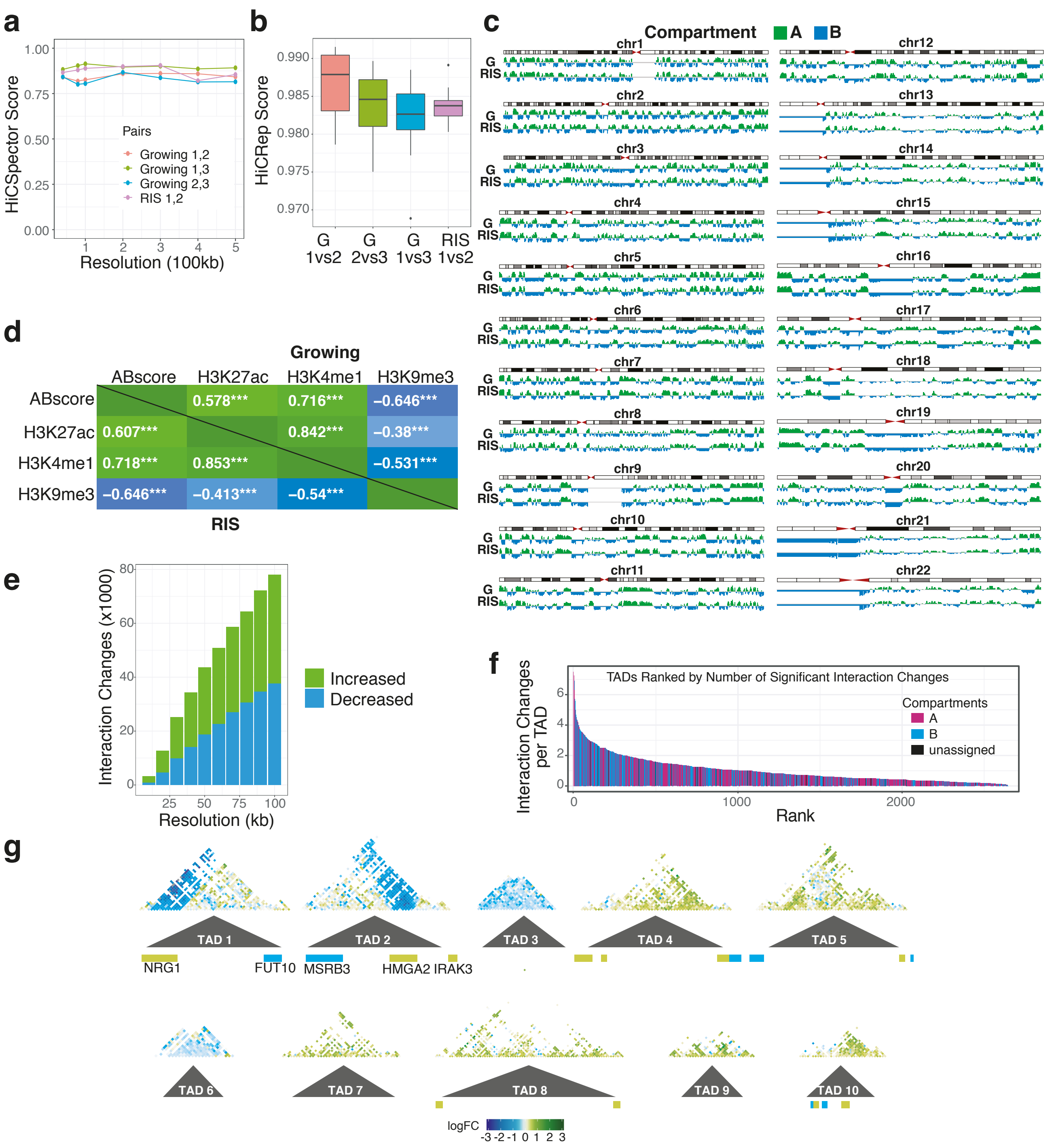

Supplementary Figure 1. A/B compartment distribution and the ranking of TADs with the most significant changes during RIS. a HiC-spector agreement scores between each pair of growing and RIS biological replicates at resolutions between 10 kb and 200 kb; scores are between 0 and 1, indicating poor and good agreement, respectively. **b** HiCRep pairwise agreement scores for each pair of growing and RIS replicates at 40 kb resolution, ranging between 0 and 1, where 1 indicates perfect agreement; Box plots correspond to the median, 25th to 75th percentiles, and the whiskers correspond to the 10th to 90th percentiles. **c** Distribution across each chromosome of the principal component corresponding to A/B compartments (based on correlation with H3K4me1 signal) from PCA performed on growing (G) and RIS Hi-C libraries; positive values (green) mark the A compartment, whereas negative values (blue) mark the B compartment. **d** Correlation between A/B compartment score and THOR-normalized ChIP-seq signal for H3K4me1, H3K27ac and H3K9me3 marks, ***$p \leq 0.001$. **e** Number of significant interaction changes determined genome-wide using information from all replicates of growing and RIS, at resolutions between 10 and 100 kb. **f** TADs ranked by the total number of significant differential interactions occurring within them, normalized for their size, and coloured by colocalization of each TAD with the A or B compartment. **g** The top 10 TADs with the most interaction changes during RIS, represented by their interaction log-fold changes at 20 kb resolution, flanked by 100 kb on either side of the TAD and the actual TAD position plotted below as grey triangles. Differentially expressed genes are marked below the TADs, with green and blue segments corresponding to up-regulated and down-regulated genes, respectively.

Supplementary Fig. 2_Olan et al.

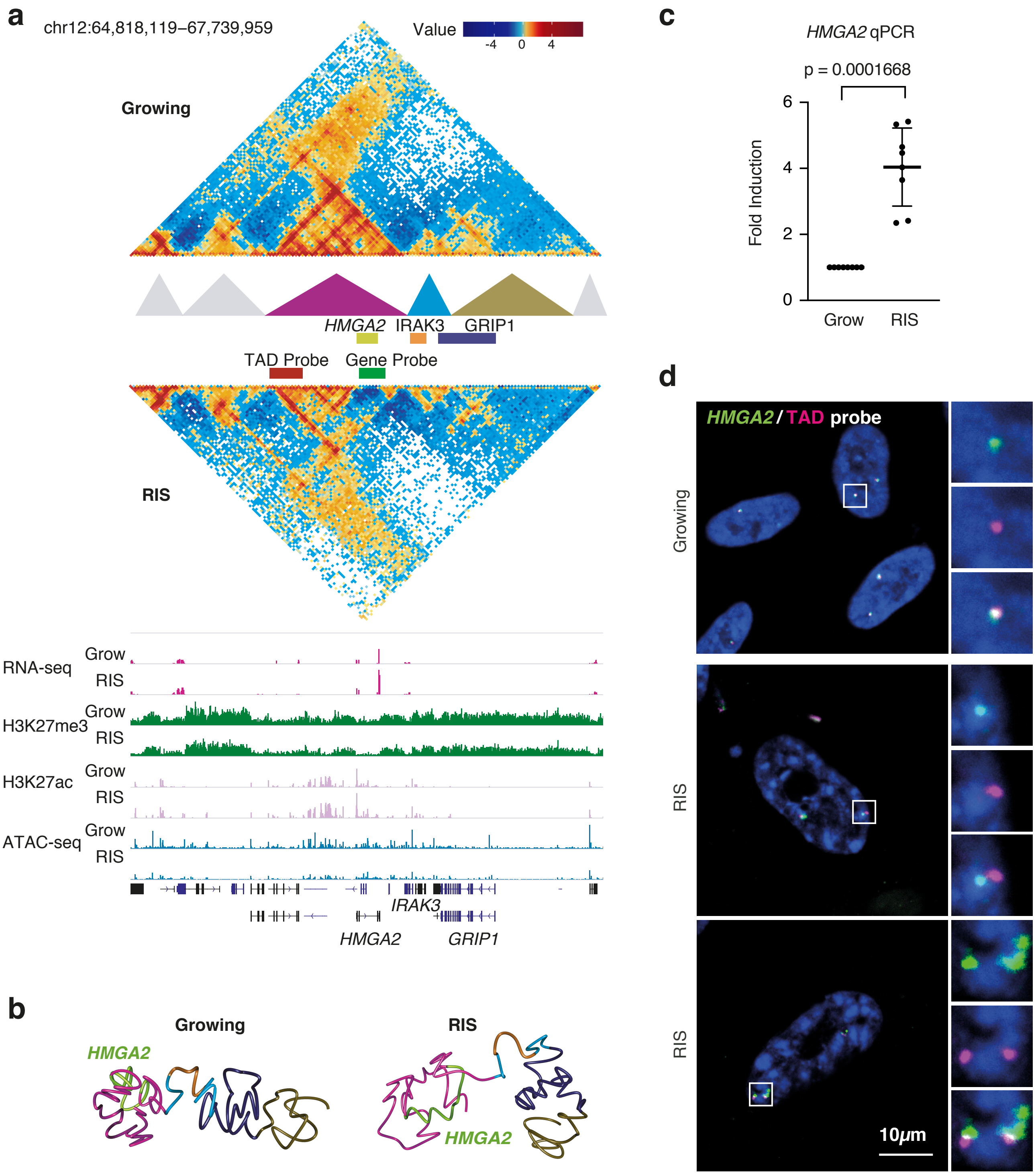

Supplementary Figure 2. *HMGA2* detachment from its H3K27me3 TAD concomitant with its up-regulation during RIS. a Hi-C interaction matrices in Growing and RIS IMR90 cells at 20 kb resolution, encompassing the *HMGA2* gene and surrounding TADs. Representative matching RNA-seq (TMM-normalized) and tracks of THOR-normalized ChIP-seq (H3K27me3 and H3K27ac) and ATAC-seq are aligned below. **b** TADbit three-dimensional modelling of the three TADs marked by the coloured triangles in (a), colouring the *HMGA2*, *IRAK3* and *GRIP1* genes as well as the TADs they belong to. **c** qPCR validation of the up-regulation of *HMGA2* in RIS compared to growing cells. Values are mean +- SD, across n = 8 biological replicates. Statistical significance was calculated using two-sided Student's t-test, p=0.0002. **d** Representative DNA-FISH images for the indicated conditions, using FISH probes corresponding to the *HMGA2* locus (gene-probe, green) and a nearby H3K27me3 region within the same TAD (TAD-probe, magenta), as per the locations marked in (a). The regions indicated by rectangles are magnified. See Fig. 1e for quantification.

Supplementary Fig. 3_Olan et al.

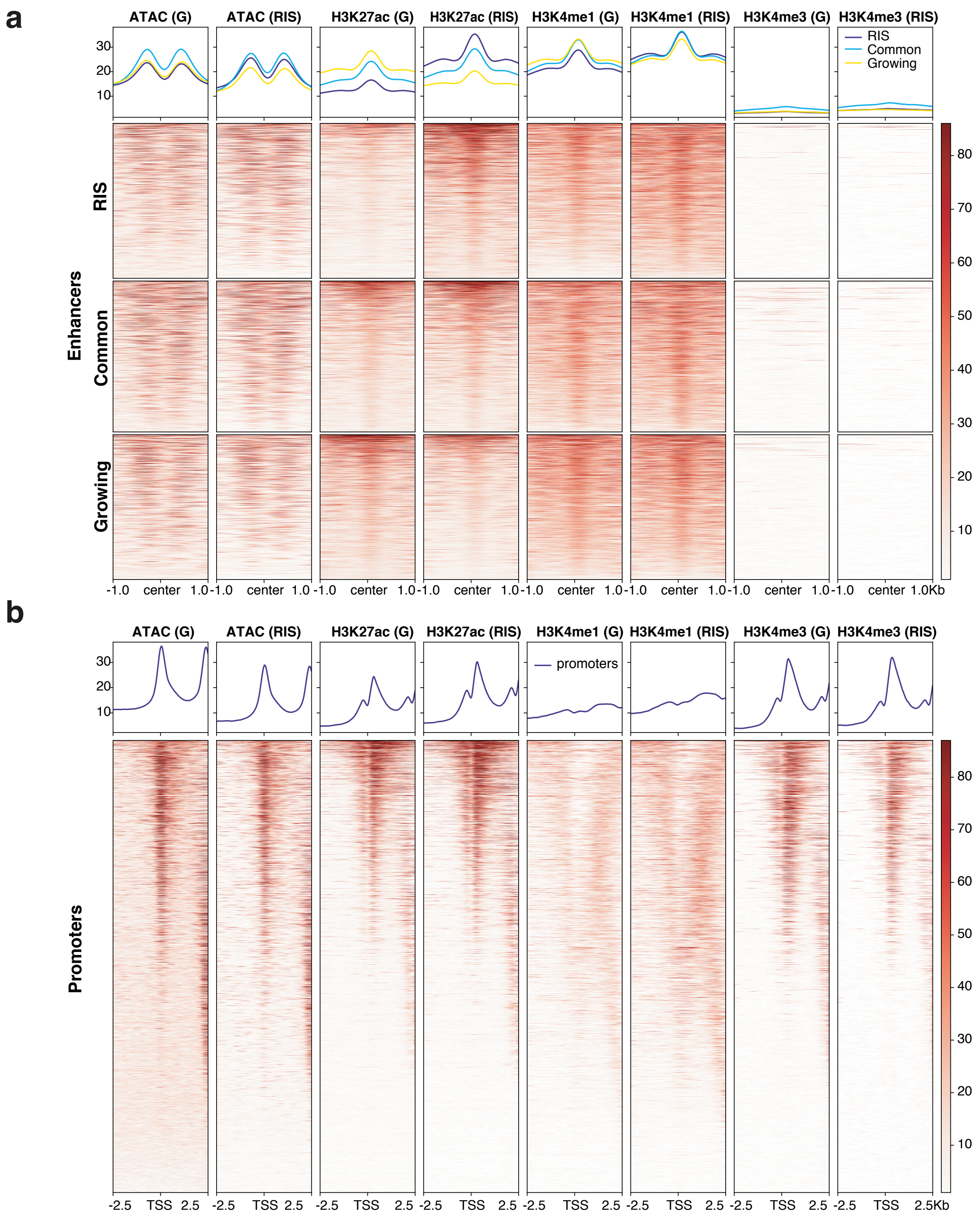

Supplementary Figure 3. Epigenetic characterization of enhancers and promoters using ATAC-seq and H3K27ac, H3K4me1 and H3K4me3 ChIP-seq signal. a, b Heatmaps of the indicated ChIP-seq and ATAC-seq for enhancer (a) and promoter (b) regions. Enhancers were defined by H3K27ac peaks which also have high H3K4me1 and low H3K4me3 ChIP-seq signal. Enhancer data are split by regions common between growing (G) and RIS and specific to each condition. Promoters were defined as 5 kb regions around the TSS of every protein-coding gene.

Supplementary Fig. 4_Olan et al.

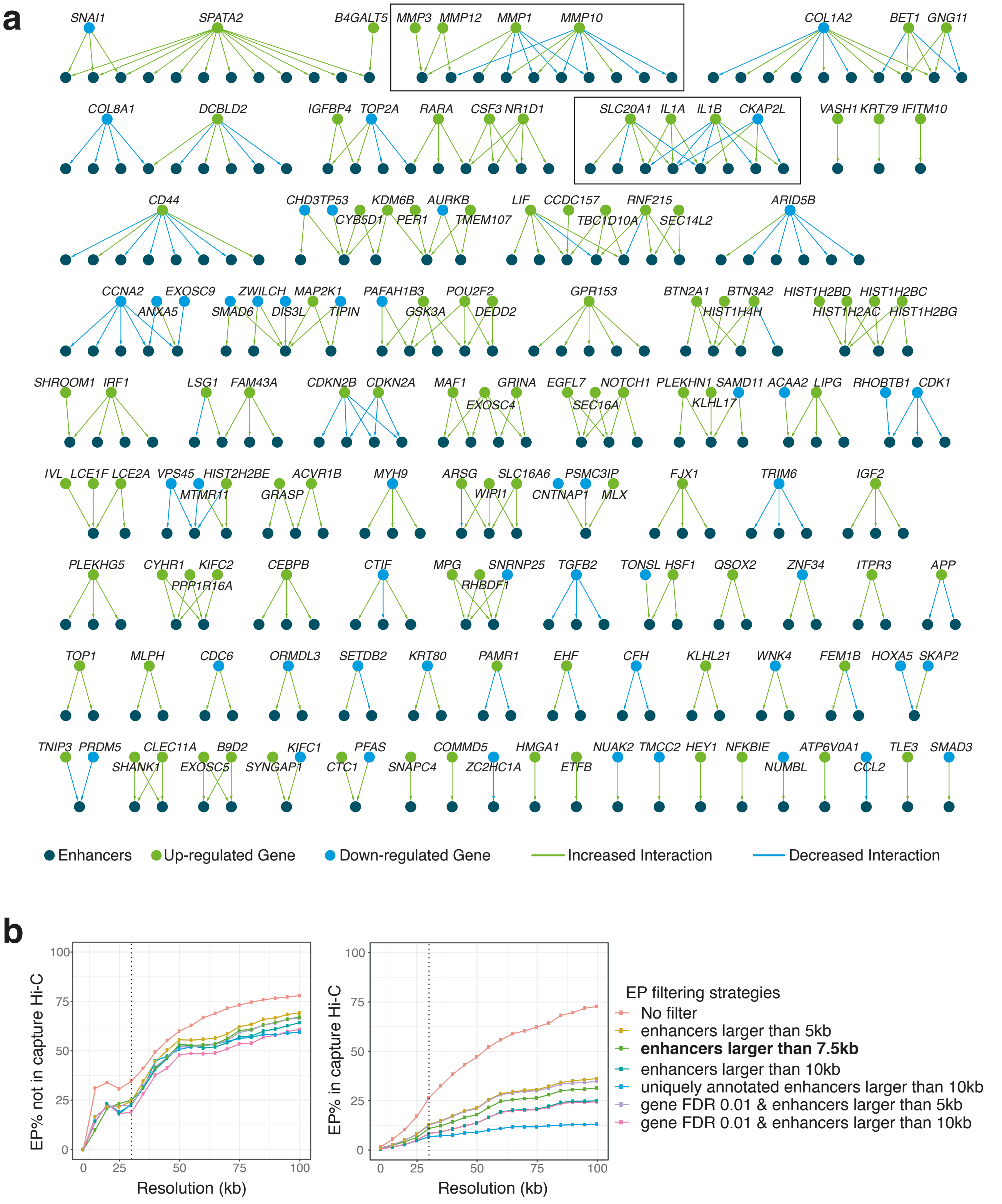

Supplementary Figure 4. Enhancer-Promoter (EP) Network from capture Hi-C. a Differential EP interactions network based on annotated cHi-C significant interaction changes during RIS at HindIII resolution. **b** Comparison between EP interactions annotated from genome-wide Hi-C analysis at resolutions between 10 kb and 100 kb, and EP interactions annotated from cHi-C (represented in **a**), filtered with different strategies such as enhancer size or gene expression change. Left, percentage of EP annotated from Hi-C but not from cHi-C ('false positives') in the captured regions. Right, percentage of EP annotated from Hi-C as well as from cHi-C ('true positives'). The filtering strategy highlighted that 'enhancers larger than 7.5 kb' and 'bin sizes smaller than 30 kb' minimise the EP annotated from Hi-C but not from cHi-C and maximise the ones annotated in both Hi-C and cHiC.

Supplementary Fig. 5_Olan et al.

a

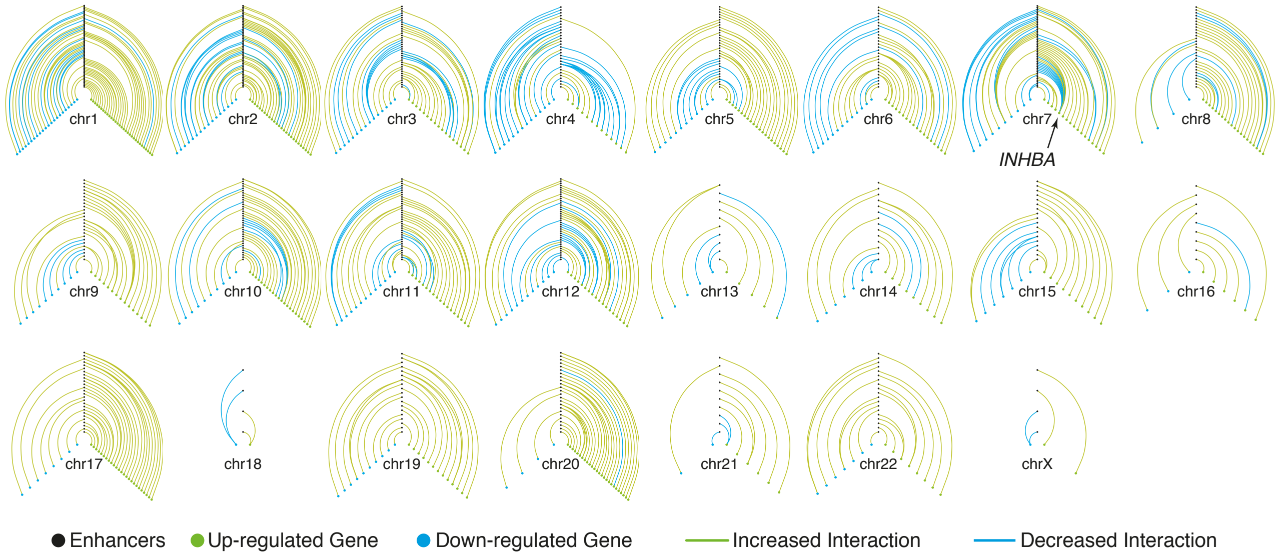

b

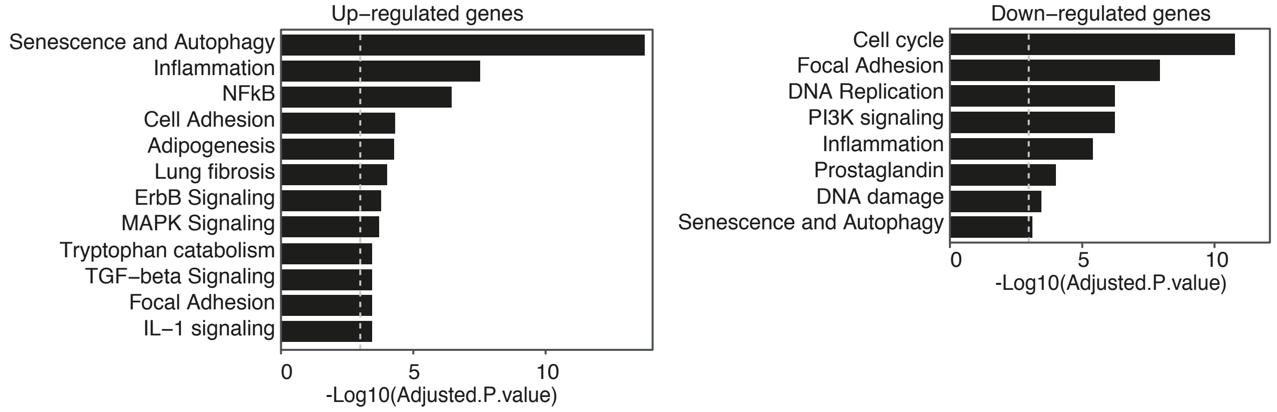

Supplementary Figure 5. Genome-wide Hi-C EP differential network. a EP network using the filtering strategy based on EP changes annotated from cHi-C (Supplementary Fig. 4b, enhancers larger than 7.5 kb and resolution higher than 30 kb, i.e. bin sizes smaller than 30 kb) on each chromosome, with the vertical axis (black dots) representing enhancers and the right and the left axes corresponding to up-regulated (green dots) and down-regulated (blue dots) genes, respectively. Green and blue arcs represent increased and decreased interaction, respectively. The *INHBA* gene is highlighted, representing the largest component in the network. **b** Gene enrichment with EnrichR against the WikiPathways 2019 database of up-regulated (left) and down-regulated (right) genes in the genome-wide Hi-C EP differential network. The dotted grey line corresponds to 0.05 adjusted p-value, the selected threshold for significant enrichment. Terms were manually simplified and obvious redundancy was removed.

a

b

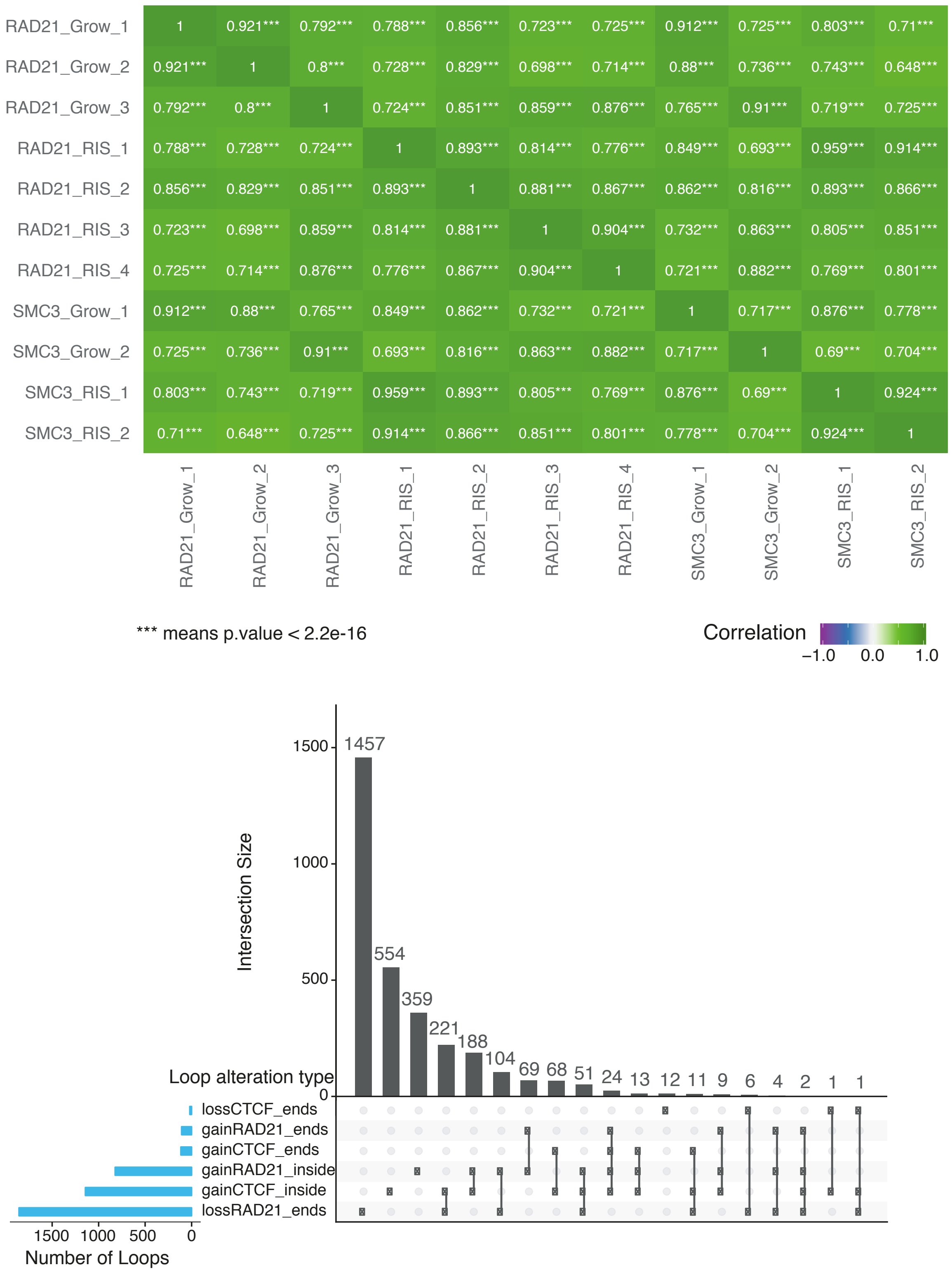

Supplementary Figure 6. Distribution of altered cohesin and CTCF binding in loops. a Agreement between RAD21 and SMC3 ChIP-seq. Pairwise Pearson correlation values between every replicate of RAD21 and SMC3 THOR-normalized ChIP-seq signal in growing and RIS. ***p ≤ 0.001. Statistical significance was calculated using a Pearson correlation test. **b** UpSet plot of 3,154 loops that exhibited differential binding of cohesin and/or CTCF at loop anchors and/or loop domains.

Supplementary Fig. 7_Olan et al.

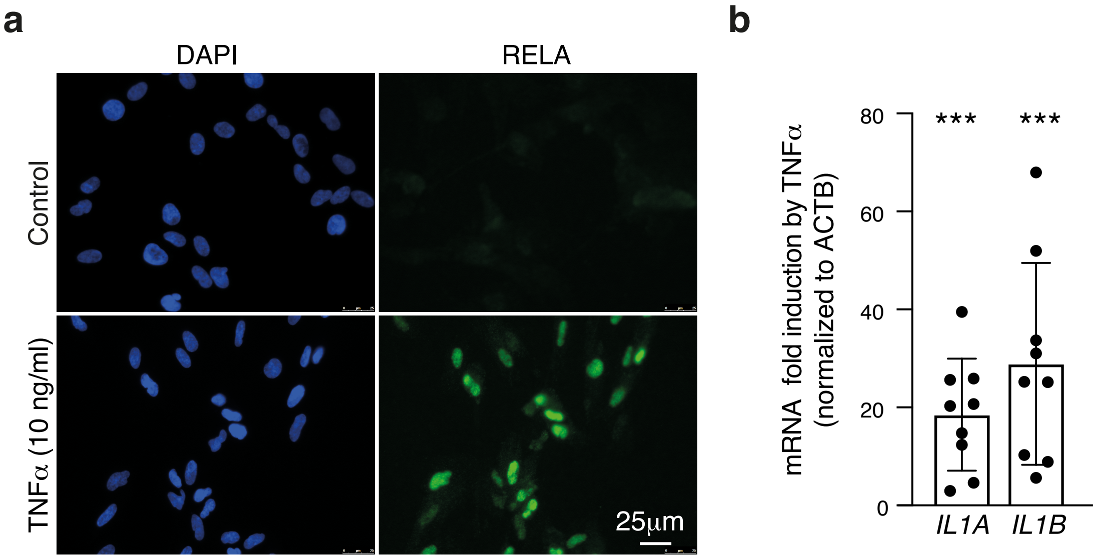

Supplementary Figure 7. Acute response to TNFα treatment in IMR90 cells: Cells were treated with 10 ng/ml TNFα for 1 hour before harvesting. **a** Immunofluorescence experiments showing nuclear localization of RELA (a major NF-κB component) with TNFα treatment. **b** qPCR showing up-regulation of *IL1A* and *IL1B* with TNFα treatment relative to untreated control cells. Values are mean +- SD, across n = 9 biological replicates. ***p ≤ 0.001 (*IL1A*: p=0.0003, *IL1B*: p=0.0009). Statistical significance was calculated using two-sided Student's t-test.

Supplementary Fig. 8_Olan et al.

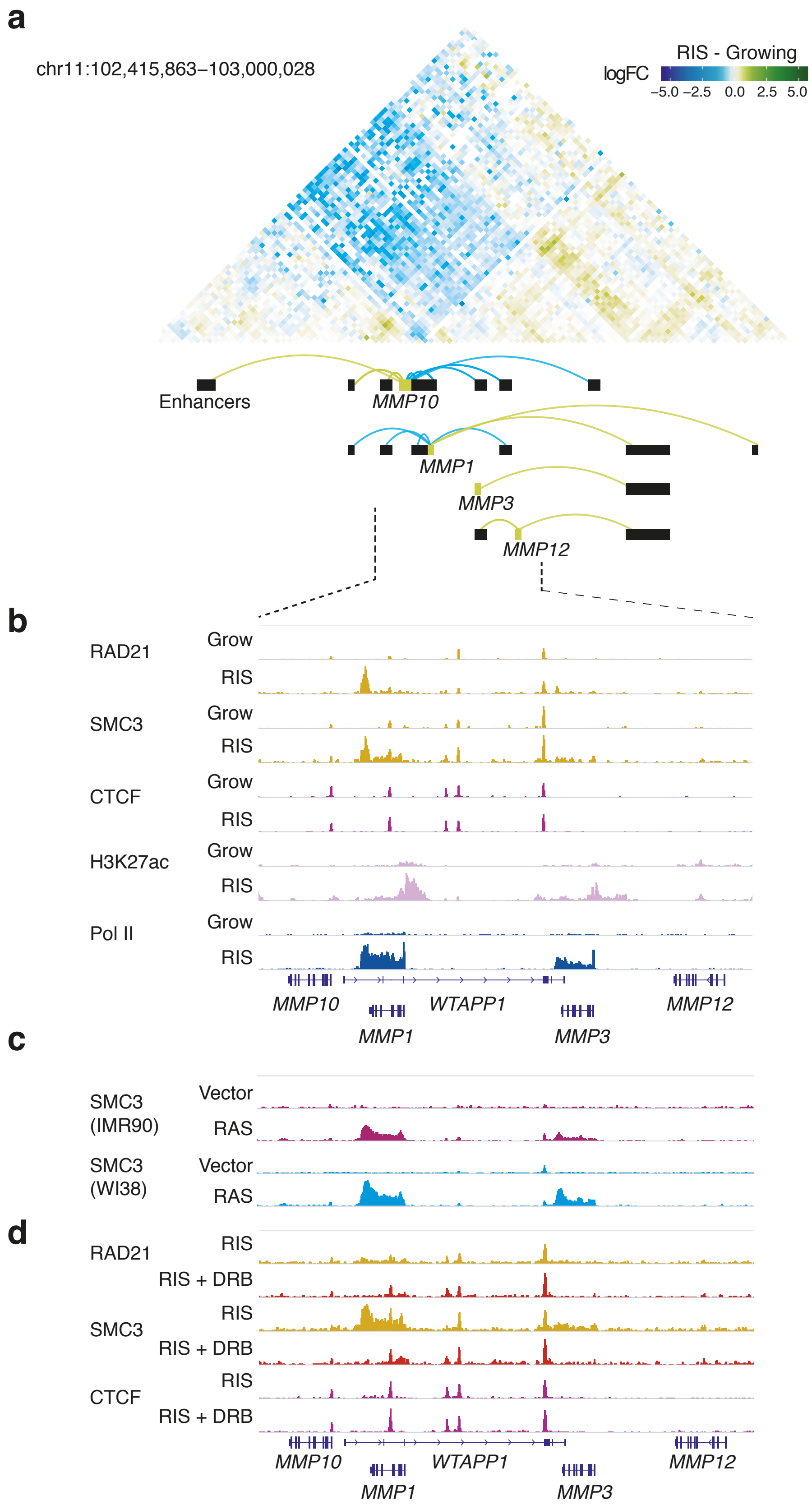

Supplementary Figure 8. Interaction changes during RIS at the *MMP* locus suggesting increased spatial separation around *MMP1*. a cHi-C differential interaction matrix (*HindIII* resolution) at the *MMP* locus, consisting of the promoters of *MMP10, MMP1, MMP3*, *MMP12* and associated enhancers. Arcs represent significant alterations in EP contacts (the green and blue arcs correspond to increased and decreased interactions, respectively). All *MMPs* shown were upregulated during RIS. **b** Representative THOR-normalized ChIP-seq tracks for the indicated factors at the *MMP* locus marked with dotted lines in (a), in growing and RIS. **c** Representative SMC3 ChIP-seq tracks at the *MMP* locus in RIS IMR90 and WI38 cells via constitutive expression of oncogenic *HRAS-G12V* and matched growing controls (empty vector). **d** Representative RAD21, SMC3 and CTCF ChIP-seq tracks in RIS with and without DRB treatment (transcription elongation inhibitor) at the *MMP* locus.

Supplementary Fig. 9_Olan et al.

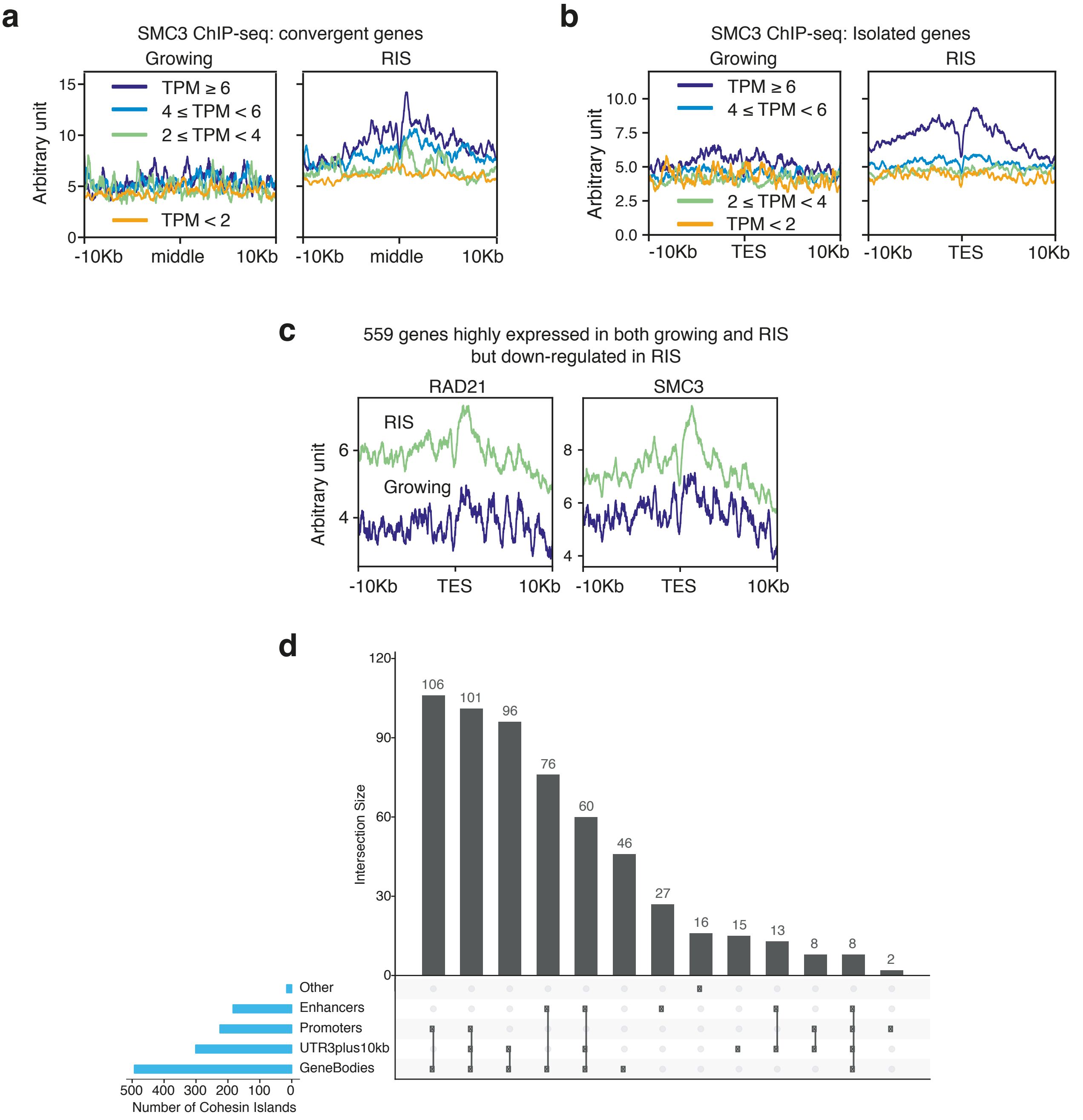

Supplementary Figure 9. Increased cohesin binding correlates with high gene expression in RIS but not growing cells. a, b Cohesin (THOR-normalized SMC3 ChIP-seq signal) distribution at the 3' end of genes in RIS cells, grouped by log-transcripts-per-million (TPM) expression at convergent genes (overlapping extended 3' ends) (a) and isolated genes (no overlap with other genes) (b). middle, middle points between the converging 3' ends; TES, transcriptional end site. In the case of convergent genes, both genes in the pair were in the same expression category. **c** RAD21 and SMC3 ChIP-seq signal at the 3' end of 559 genes which are highly expressed in growing and RIS but more highly in growing. **d** UpSet plot showing the distribution of cohesin islands over genomic regions of interest.

Supplementary Fig. 10_Olan et al.

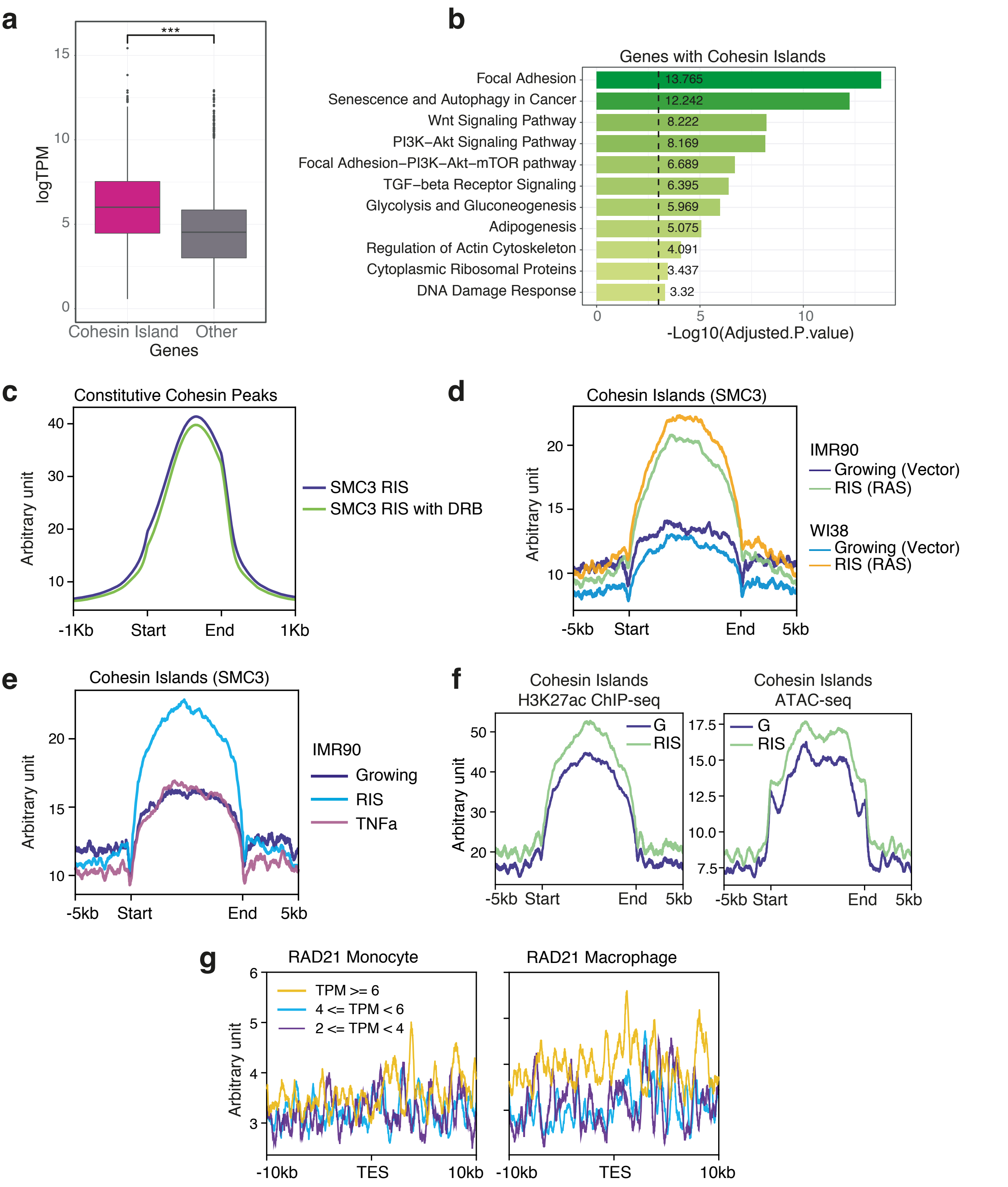

Supplementary Figure 10. Characterization of genes associated with cohesin islands. a Expression (log-transcripts-per-million) of genes with (n=614) and without (n=11,248) cohesin islands. $p < 2.2e-16$. Statistical significance calculated using a two-sided Student's t-test; Box plots correspond to the median, 25th to 75th percentiles, and the whiskers correspond to the 10th to 90th percentiles. **b** Gene enrichment pathways of genes with cohesin islands against WikiPathways 2019 (as part of the EnrichR[67] tool); dotted line corresponds to the significance threshold, 0.05 p-adjusted. **c** Profiles of constitutive cohesin peaks, defined as cohesin (SMC3 ChIP-seq) peaks which overlap CTCF peaks, in RIS cells with and without DRB treatment. **d** SMC3 ChIP-seq signal in constitutive HRAS-G12V-induced senescence and matched controls (empty vector), in IMR90 and WI38 cells, over the cohesin islands identified from RIS compared with RIS with DRB treatment. **e** SMC3 ChIP-seq signal of RIS, TNFα-treated and matched control IMR90 cells over the cohesin islands. **f** H3K27ac ChIP-seq (left) and ATAC-seq (right) profiles of cohesin islands in growing (G) and RIS IMR90 cells. **g** Profiles of RAD21 ChIP-seq (obtained from the study by Heinz et al.[45]) around the 3' end of genes grouped by expression levels (RNA-seq from Phanstiel et al.[44]) in THP-1 monocytes and PMA-induced macrophages.
