# Supplementary material for: Transcription-dependent cohesin repositioning rewires chromatin loops in cellular senescence
Source: Nat Commun. 2020 Nov 27;11:6049. doi: 10.1038/s41467-020-19878-4 (PMC7695716; doi:10.1038/s41467-020-19878-4)
Supplement: Supplementary file 3 — Description of Additional Supplementary Files [file 41467_2020_19878_MOESM3_ESM.docx]

Supplementary Data Description

**Supplementary Data 1.** List of captured regions with hg19 coordinates

**Supplementary Data 2.** Number of valid reads aligned from each Hi-C and cHi-C library, as well as the number of reads corresponding to various types of artefacts

**Supplementary Data 3.** The coordinates of the top 10 TADs with the most interaction changes during RIS in IMR90 cells

**Supplementary Data 4.** RIS-associated up-regulated genes, which also show reduced interactions with H3K27me3 regions

**Supplementary Data 5.** Enhancer-promoter differential contacts from cHi-C (*HindIII* resolution) and Hi-C (at different resolutions)

**Supplementary Data 6.** Genes with cohesin islands in RIS IMR90 cells

**Supplementary Data 7.** List of primers used

**Supplementary Data 8.** Source data. Quantification of the average FISH distances per cell used for Fig. 1e, data values used for Supplementary Fig. 2c and 7b
