# Supplementary material for: Transcription-dependent cohesin repositioning rewires chromatin loops in cellular senescence
Source: Nat Commun. 2020 Nov 27;11:6049. doi: 10.1038/s41467-020-19878-4 (PMC7695716; doi:10.1038/s41467-020-19878-4)
Supplement: Supplementary file 12 — Reporting Summary [file 41467_2020_19878_MOESM12_ESM.pdf]

nature research

Corresponding author(s): Masashi Narita, Peter Fraser

Last updated by author(s): 27/10/2020

Reporting Summary

Nature Research wishes to improve the reproducibility of the work that we publish. This form provides structure for consistency and transparency in reporting. For further information on Nature Research policies, see our Editorial Policies and the Editorial Policy Checklist.

Statistics

For all statistical analyses, confirm that the following items are present in the figure legend, table legend, main text, or Methods section.

n/a	Confirmed	
☐	☒	The exact sample size (*n*) for each experimental group/condition, given as a discrete number and unit of measurement
☐	☒	A statement on whether measurements were taken from distinct samples or whether the same sample was measured repeatedly
☐	☒	The statistical test(s) used AND whether they are one- or two-sided<br>*Only common tests should be described solely by name; describe more complex techniques in the Methods section.*
☒	☐	A description of all covariates tested
☐	☒	A description of any assumptions or corrections, such as tests of normality and adjustment for multiple comparisons
☐	☒	A full description of the statistical parameters including central tendency (e.g. means) or other basic estimates (e.g. regression coefficient) AND variation (e.g. standard deviation) or associated estimates of uncertainty (e.g. confidence intervals)
☐	☒	For null hypothesis testing, the test statistic (e.g. *F*, *t*, *r*) with confidence intervals, effect sizes, degrees of freedom and *P* value noted<br>*Give P values as exact values whenever suitable.*
☒	☐	For Bayesian analysis, information on the choice of priors and Markov chain Monte Carlo settings
☒	☐	For hierarchical and complex designs, identification of the appropriate level for tests and full reporting of outcomes
☐	☒	Estimates of effect sizes (e.g. Cohen's *d*, Pearson's *r*), indicating how they were calculated

Our web collection on statistics for biologists contains articles on many of the points above.

Software and code

Policy information about availability of computer code

Data collection

No software was used.

Data analysis

Code availability. Custom scripts used for enhancer-promoter annotation and filtering THOR differential binding output were uploaded to the OSF public repository (https://osf.io/xajd3/?view_only=6860fe4b8421475485b7e251d735db58). A package for visualization of Hi-C matrices is also available on GitLab (https://gitlab.com/ilyco/hicvizr).

We used the following software for data analysis:
HiC-Pro (2.10.0
TADbit (0.4.2)
R (3.4.4)
python (2.7.12)
MACS2 (2.1.1.20160309)

THOR (0.11.4)
FastQC (0.11.5)
samtools (1.6 (using htslib 1.6))
deeptools (2.5.4)
bowtie2 (2.3.0 64bit)
STAR (2.5.3a)
subread (1.5.2)
HiC-Spector (1.0)
HiCRep (1.2.0)

We used the following R packages for data analysis and visualization (mentioned in the Methods section):
R CRAN rgl (0.100.19)
R CRAN ggplot2 (3.1.0)
R CRAN enrichR (1.0)
R CRAN ggsignif (0.4.0)
R CRAN package NMI (2.0)
R Bioconductor diffHic (1.10.0)
R Bioconductor edgeR (3.20.9)
R Bioconductor ChIPQC (1.14.0)

Analysis of FISH signal was performed using scikit-image (https://scikit-image.org, 0.14.1).

For manuscripts utilizing custom algorithms or software that are central to the research but not yet described in published literature, software must be made available to editors and reviewers. We strongly encourage code deposition in a community repository (e.g. GitHub). See the Nature Research guidelines for submitting code & software for further information.

Data

Policy information about availability of data

All manuscripts must include a data availability statement. This statement should provide the following information, where applicable:
- Accession codes, unique identifiers, or web links for publicly available datasets
- A list of figures that have associated raw data
- A description of any restrictions on data availability

Data availability. Hi-C and cHi-C data in growing and senescent IMR90 cells, as well as ChIP-seq data in IMR90 and WI38 human diploid fibroblasts in the growing (with and without TNF treatment) and RIS (with and without DRB treatment) conditions were deposited in the Gene Expression Omnibus: GSE135093 [https://www.ncbi.nlm.nih.gov/geo/query/acc.cgi?acc=GSE135093]. Publicly available data in growing and senescent IMR90 cells were reanalysed from our previous studies: H3K4me3 and H3K27me3 ChIP-seq from Chandra et al.25 (GSE38448, [https://www.ncbi.nlm.nih.gov/geo/query/acc.cgi?acc=GSE38448]), H3K27ac ChIP-seq and ATAC-seq from Parry et al.27 (GSE103590, [https://www.ncbi.nlm.nih.gov/geo/query/acc.cgi?acc=GSE103590]), RNA-seq data from Hoare et al.26 (GSE72404, [https://www.ncbi.nlm.nih.gov/geo/query/acc.cgi?acc=GSE72404]). The following external datasets were reanalysed: RAD21 and CTCF ChIP-seq in monocyte (THP-1) and macrophage (PMA-induced) controls from Heinz et al.45 (GSE103477, [https://www.ncbi.nlm.nih.gov/geo/query/acc.cgi?acc=GSE103477]), RNA-seq and Hi-C in monocyte (THP-1) and macrophage (PMA-induced) controls from Phanstiel et al.44 (GSE96800, [https://www.ncbi.nlm.nih.gov/geo/query/acc.cgi?acc=GSE96800], PRJNA385337, [https://www.ncbi.nlm.nih.gov/bioproject/385337]), RNA-seq and Hi-C in IMR90 cells with or without TNF treatment from Jin et al.15 (GSE43070, [https://www.ncbi.nlm.nih.gov/geo/query/acc.cgi?acc=GSE43070]), Hi-C data in growing and senescence WI38 cells from Chandra et al.7 (PRJEB8073, [https://www.ebi.ac.uk/ena/browser/view/PRJEB8073]). All other relevant data supporting the key findings of this study are available within the article and its Supplementary Information files or from the corresponding author upon reasonable request. Source data are provided with this paper. A reporting summary for this Article is available as a Supplementary Information file.

Field-specific reporting

Please select the one below that is the best fit for your research. If you are not sure, read the appropriate sections before making your selection.

☒ Life sciences ☐ Behavioural & social sciences ☐ Ecological, evolutionary & environmental sciences

For a reference copy of the document with all sections, see nature.com/documents/nr-reporting-summary-flat.pdf

Life sciences study design

All studies must disclose on these points even when the disclosure is negative.

Sample size	Sample sizes for ChIP-seq and Hi-C experiments were determined by comparison with previous studies (see below) and by taking into consideration the fact that we are studying one condition (RAS-induced senescence) against control (growing, proliferating cells). The dynamic range of DNA binding of the proteins studied (CTCF, RAD21, SMC3) appeared saturated based on the number of common peaks between replicates and on the Pearson correlation coefficients between normalised signal tracks. The number of Hi-C and cHi-C replicates was assessed based on their good agreement (as determined with two independent methods) and the cis/trans interactions ratio, which was similar to other studies.<br><br>Experience of the authors performing Hi-C and ChIP-seq experiments in previous studies, as well as their experience in developing Capture HiC (cHi-C), allowed us to confidently select the number of replicates and library sizes for each of our experiments. Please refer to the following studies which use two Hi-C/cHi-C replicates per condition and similar library sizes per sample:<br><br>Thiecke MJ, Wutz G, Muhar M, Tang W, Bevan S, Malysheva V, Stocsits R, Neumann T, Zuber J, Fraser P, Schoenfelder S, Peters JM, Spivakov M (2020)<br>Cohesin-dependent and independent mechanisms support chromosomal contacts between promoters and enhancers<br>Cell Reports 32:107929<br><br>Schoenfelder S, Mifsud B, Senner CE, Todd CD, Chrysanthou S, Darbo E, Hemberger M, Branco MR (2018)<br>Divergent wiring of repressive and active chromatin interactions between mouse embryonic and trophoblast lineages

Nature Communications 9:4189

Koohy H, Bolland DJ, Matheson LS, Schoenfelder S, Stellato C, Dimond A, Várnai C, Chovanec P, Chessa T, Denizot J, Manzano Garcia R, Wingett SW, Freire-Pritchett P, Nagano T, Hawkins P, Stephens L, Elderkin S, Spivakov M, Fraser P, Corcoran AE, Varga-Weisz PD (2018)
Genome organization and chromatin analysis identify transcriptional downregulation of insulin-like growth factor signaling as a hallmark of aging in developing B cells
Genome Biology 19: 126

Novo CL, Javierre BM, Cairns J, Segonds-Pichon A, Wingett SW, Freire-Pritchett P, Furlan-Magaril M, Schoenfelder S, Fraser P, Rugg-Gunn PJ (2018)
Long-range enhancer interactions are prevalent in mouse embryonic stem cells and are reorganized upon pluripotent state transition
Cell Reports 22: 2615-2627

Comoglio F, Park HJ, Schoenfelder S, Barozzi I, Bode D, Fraser P, Green AR (2018)
Thrombopoietin signaling to chromatin elicits rapid and pervasive epigenome remodeling within poised chromatin architectures
Genome Research 28: 295-309

Freire-Pritchett P, Schoenfelder S, Várnai C, Wingett SW, Cairns J, Collier AJ, García-Vílchez R, Furlan-Magaril M, Osborne CS, Fraser P, Rugg-Gunn PJ, Spivakov M (2017)
Global reorganisation of cis-regulatory units upon lineage commitment of human embryonic stem cells
eLife 6. pii: e21926

McGovern A, Schoenfelder S, Martin P, Massey J, Duffus K, Plant D, Yarwood A, Pratt AG, Anderson AE, Isaacs JD, Diboll J, Thalayasingam N, Ospelt C, Barton A, Worthington J, Fraser P, Eyre S, Orozco G (2016)
Capture Hi-C identifies a novel causal gene, IL20RA, in the pan-autoimmune genetic susceptibility region 6q23.
Genome Biology 17:212.

Wilson NK, Schoenfelder S, Hannah R, Sánchez Castillo M, Schütte J, Ladopoulos V, Mitchelmore J, Goode DK, Calero-Nieto FJ, Moignard V, Wilkinson AC, Jimenez-Madrid I, Kinston S, Spivakov M, Fraser P, Göttgens B (2016)
Integrated genome-scale analysis of the transcriptional regulatory landscape in a blood stem/progenitor cell model.
Blood. Jan 25. pii: blood-2015-10-677393.

Schoenfelder S, Sugar R, Dimond A, Javierre BM, Armstrong H, Mifsud B, Dimitrova E, Matheson L, Tavares-Cadete F, Jurkowski W, Segonds-Pichon A, Wingett S, Furlan-Magaril M, Tabbada K, Andrews S, Herman B, LeProust E, Osborne CS, Koseki H, Fraser P, Luscombe NM, Elderkin S (2015)
Polycomb repressive complex PRC1 spatially constrains the mouse embryonic stem cell genome
Nature Genetics 47: 1179-86

Schoenfelder S, Furlan-Magaril M, Mifsud B, Tavares-Cadete F, Sugar R, Javierre BM, Nagano T, Katsman Y, Sakthidevi M, Wingett S, Dimitrova E, Dimond A, Edelman LB, Elderkin S, Tabbada K, Darbo E, Andrews S, Herman B, Higgs A, LeProust E, OsborneCS, Mitchell JA, Luscombe NM, Fraser P (2015)
The pluripotent regulatory circuitry connecting promoters to their long-range interacting elements
Genome Research 25: 582-597

Mifsud B, Tavares-Cadete F, Young AN, Sugar R, Schoenfelder S, Ferreira L, Wingett S, Andrews S, Ewels P, Herman B, Happe S, Higgs A, LeProust E, Follows GA, Fraser P, Luscombe NM, Osborne CS (2015)
High-resolution capture Hi-C to map long-range promoter contacts in human cells
Nature Genetics 47: 598-606

Similarly, we chose a minimum of two replicates per condition for each ChIP-seq experiment, with more the four replicates for our main results regarding cohesin DNA binding (described in depth in the sections below dedicated to ChIP-seq), based on our previous studies performing ChIP-seq in human fibroblasts:

Chandra, T. et al. Independence of Repressive Histone Marks and Chromatin Compaction during Senescent Heterochromatic Layer Formation. Mol. Cell 47, 203–214 (2012).

Sadaie, M. et al. Redistribution of the Lamin B1 genomic binding profile affects rearrangement of heterochromatic domains and SAHF formation during senescence. Genes Dev. 27, 1800–8 (2013).

Parry, A. J. et al. NOTCH-mediated non-cell autonomous regulation of chromatin structure during senescence. Nat. Commun. 9, 1-15 (2018).

Data exclusions

One of the RAS-induced Senescence Hi-C samples was excluded due to lower number of available valid reads and low complexity, which rendered its comparison to control samples inadequate and thus we included two Hi-C RAS-induced Senescence replicates and three Growing replicates. No other data exclusions were performed as all other experiments (cHi-C, all ChIP-seq) were replicated with success.

Replication	Our Hi-C dataset consists of three Growing control replicates and two RAS-induced Senescence replicates, as well as two capture Hi-C Growing replicates and two capture Hi-C RAS-induced Senescence replicates. We used HiC-Spector and HiCRep in order to check the similarity between Hi-C samples and good agreement was reported. For ChIP-seq experiments, we checked agreement between replicates in terms of number of common peaks detected and overall Pearson correlation between normalized signal (with subtracted input), calculated genome-wide using 10kb bins. The number of ChIP-seq replicates for each condition is described in detail below. qPCR experiments were performed to confirm the up-regulation of HMGA2, IL1A and IL1B with the following number of replicates: 8 replicates (8 growing, 8 senescence) for HMGA2, and 9 replicates for growing and 9 senescence replicates for IL1A and IL1B. FISH experiments for NRG1 and HMGA2 genes and nearby genomic regions were performed in two replicates of Growing and two replicates of senescence.<br><br>All attempts at replication were successful, except for one of the Hi-C samples which exhibited low complexity and was removed from further analysis.
Randomization	Only two experimental groups are analysed mainly throughout our study: control and senescence. All the comparisons we performed were pairwise between control and senescent cells or between control and other treatment (TNFa/DRB). We prioritized handling samples in ways which ensure no accidental mislabeling or switching can occur and batch effects are minimized. The readout from all the experiments was sequencing.
Blinding	Blinding is not applicable in our case because no grouping was present besides the two conditions studied, which have to be prepared in different ways and cannot be mislabeled.

Reporting for specific materials, systems and methods

We require information from authors about some types of materials, experimental systems and methods used in many studies. Here, indicate whether each material, system or method listed is relevant to your study. If you are not sure if a list item applies to your research, read the appropriate section before selecting a response.

Materials & experimental systems

n/a	Involved in the study	
☐	☒	Antibodies
☐	☒	Eukaryotic cell lines
☒	☐	Palaeontology and archaeology
☒	☐	Animals and other organisms
☒	☐	Human research participants
☒	☐	Clinical data
☒	☐	Dual use research of concern

Methods

n/a	Involved in the study	
☐	☒	ChIP-seq
☒	☐	Flow cytometry
☒	☐	MRI-based neuroimaging

Antibodies

Antibodies used	anti-H3K27ac (Hiroshi Kimura Laboratory, clone CMA309, 10 μg/20M cells)<br>anti-H3K27me3 (Hiroshi Kimura Laboratory, clone CMA323, 5 μg/10M cells)<br>anti-CTCF (Cell Signaling Technology, 3418, lot # 1, clone D31H12, 10 μg/20M cells)<br>anti-RAD21 (Katsuhiko Shirahige Laboratory, 10 μg/20M cells)<br>anti-SMC3 (Abcam ab9263, lot # GR290533-17 and GR3221084-8, 10 μg/20M cells)<br>anti-PolII (Hiroshi Kimura Laboratory, clone C13B9, 10 μg/20M cells)<br>anti-RELA (Cell Signalling Technology, 8242, lot # 13, 1:400)<br>anti-rabbit IgG (Alexa Fluor 488, Thermo Fisher, A-11034, lot # 2069632, 1:1000)
Validation	anti-H3K27ac and anti-H3K27me3 and anti-PolII - mentioned in Kimura, H., Hayashi-Takanaka, Y., Goto, Y., Takizawa, N. & Nozaki, N. The Organization of Histone H3 Modifications as Revealed by a Panel of Specific Monoclonal Antibodies. Cell Struct. Funct. 33, 61–73 (2008).<br>anti-RAD21: mentioned in Minamino, M. et al. Esco1 Acetylates Cohesin via a Mechanism Different from That of Esco2. Curr. Biol. 25, 1694–1706 (2015).<br>anti-CTCF: Cell Signaling Technology mentions several studies which used this antibody including Liu, E. M. et al. Identification of Cancer Drivers at CTCF Insulators in 1,962 Whole Genomes. Cell Syst. 8, 446-455.e8 (2019).<br>anti-SMC3: Abcam mentions several studies which used this antibody including: Wang, T. et al. Smc3 is required for mouse embryonic and adult hematopoiesis. Exp. Hematol. 70, 70-84.e6 (2019).<br>anti-RELA: Cell Signaling Technology mentions several studies which used this antibody including Kozako T, Suzuki T, Yoshimitsu M, et al. Novel small-molecule SIRT1 inhibitors induce cell death in adult T-cell leukaemia cells. Sci Rep. 2015;5:11345. 2015 Jun 19. doi:10.1038/srep11345<br>anti-rabbit IgG (Alexa Fluor 488, Thermo Fisher cat#A-11034): Gonzalez-Perez et al. Front Cell Neurosci. 2018 May 15;12:132. doi: 10.3389/fncel.2018.00132. eCollection 2018.

Eukaryotic cell lines

Policy information about cell lines

Cell line source(s)	IMR90 (ATCC)

	WI38 (ATCC)
Authentication	Cell identity was confirmed by STR (short tandem repeats) genotyping.
Mycoplasma contamination	Cells were regularly tested for mycoplasma contamination and always found to be negative.
Commonly misidentified lines (See ICLAC register)	No commonly misidentified cell lines were used.

ChIP-seq

Data deposition

☒ Confirm that both raw and final processed data have been deposited in a public database such as GEO.

☒ Confirm that you have deposited or provided access to graph files (e.g. BED files) for the called peaks.

Data access links<br>*May remain private before publication.*	The following secure token has been created to allow review of record GSE135093 while it remains in private status: szqfkgugzxeljmt.
Files in database submission	SLX-12520.A001.HGHNMBBXX.s_7.r_1.fq.gz<br>SLX-12520.A002.HGHNMBBXX.s_7.r_1.fq.gz<br>SLX-12520.A003.HGHNMBBXX.s_7.r_1.fq.gz<br>SLX-12520.A004.HGHNMBBXX.s_7.r_1.fq.gz<br>SLX-12520.A005.HGHNMBBXX.s_7.r_1.fq.gz<br>SLX-12520.A006.HGHNMBBXX.s_7.r_1.fq.gz<br>SLX-12520.A007.HGHNMBBXX.s_7.r_1.fq.gz<br>SLX-12520.A008.HGHNMBBXX.s_7.r_1.fq.gz<br>SLX-12520.A009.HGHNMBBXX.s_7.r_1.fq.gz<br>SLX-12520.A010.HGHNMBBXX.s_7.r_1.fq.gz<br>SLX-12520.A011.HGHNMBBXX.s_7.r_1.fq.gz<br>SLX-12520.A012.HGHNMBBXX.s_7.r_1.fq.gz<br>SLX-15808.NEBNext14.H23V3BBXY.s_6.r_1.fq.gz<br>SLX-15808.NEBNext15.H23V3BBXY.s_6.r_1.fq.gz<br>SLX-15808.NEBNext16.H23V3BBXY.s_6.r_1.fq.gz<br>SLX-15808.NEBNext18.H23V3BBXY.s_6.r_1.fq.gz<br>SLX-15808.NEBNext21.H23V3BBXY.s_6.r_1.fq.gz<br>SLX-15808.NEBNext22.H23V3BBXY.s_6.r_1.fq.gz<br>SLX-15808.NEBNext23.H23V3BBXY.s_6.r_1.fq.gz<br>SLX-15808.NEBNext25.H23V3BBXY.s_6.r_1.fq.gz<br>SLX-15809.NEBNext01.H27NVBBXY.s_1.r_1.fq.gz<br>SLX-15809.NEBNext01.H55H5BBXY.s_1.r_1.fq.gz<br>SLX-15809.NEBNext03.H27NVBBXY.s_1.r_1.fq.gz<br>SLX-15809.NEBNext03.H55H5BBXY.s_1.r_1.fq.gz<br>SLX-15809.NEBNext08.H27NVBBXY.s_1.r_1.fq.gz<br>SLX-15809.NEBNext08.H55H5BBXY.s_1.r_1.fq.gz<br>SLX-15809.NEBNext10.H27NVBBXY.s_1.r_1.fq.gz<br>SLX-15809.NEBNext10.H55H5BBXY.s_1.r_1.fq.gz<br>SLX-15809.NEBNext13.H27NVBBXY.s_1.r_1.fq.gz<br>SLX-15809.NEBNext13.H55H5BBXY.s_1.r_1.fq.gz<br>SLX-15809.NEBNext16.H27NVBBXY.s_1.r_1.fq.gz<br>SLX-15809.NEBNext16.H55H5BBXY.s_1.r_1.fq.gz<br>SLX-15809.NEBNext18.H27NVBBXY.s_1.r_1.fq.gz<br>SLX-15809.NEBNext18.H55H5BBXY.s_1.r_1.fq.gz<br>SLX-15809.NEBNext22.H27NVBBXY.s_1.r_1.fq.gz<br>SLX-15809.NEBNext22.H55H5BBXY.s_1.r_1.fq.gz<br>SLX-18021.NEBNext01.H7TY5DRXX.s_2.r_1.fq.gz<br>SLX-18021.NEBNext01.H7TY5DRXX.s_2.r_2.fq.gz<br>SLX-18021.NEBNext01.HCLNVDRXX.s_2.r_1.fq.gz<br>SLX-18021.NEBNext01.HCLNVDRXX.s_2.r_2.fq.gz<br>SLX-18021.NEBNext02.H7TY5DRXX.s_2.r_1.fq.gz<br>SLX-18021.NEBNext02.H7TY5DRXX.s_2.r_2.fq.gz<br>SLX-18021.NEBNext02.HCLNVDRXX.s_2.r_1.fq.gz<br>SLX-18021.NEBNext02.HCLNVDRXX.s_2.r_2.fq.gz<br>SLX-18021.NEBNext03.H7TY5DRXX.s_2.r_1.fq.gz<br>SLX-18021.NEBNext03.H7TY5DRXX.s_2.r_2.fq.gz<br>SLX-18021.NEBNext03.HCLNVDRXX.s_2.r_1.fq.gz<br>SLX-18021.NEBNext03.HCLNVDRXX.s_2.r_2.fq.gz<br>SLX-18021.NEBNext04.H7TY5DRXX.s_2.r_1.fq.gz<br>SLX-18021.NEBNext04.H7TY5DRXX.s_2.r_2.fq.gz

SLX-18021.NEBNext04.HCLNVDRXX.s_2.r_1.fq.gz
SLX-18021.NEBNext04.HCLNVDRXX.s_2.r_2.fq.gz
SLX-18021.NEBNext05.H7TY5DRXX.s_2.r_1.fq.gz
SLX-18021.NEBNext05.H7TY5DRXX.s_2.r_2.fq.gz
SLX-18021.NEBNext05.HCLNVDRXX.s_2.r_1.fq.gz
SLX-18021.NEBNext05.HCLNVDRXX.s_2.r_2.fq.gz
SLX-18021.NEBNext06.H7TY5DRXX.s_2.r_1.fq.gz
SLX-18021.NEBNext06.H7TY5DRXX.s_2.r_2.fq.gz
SLX-18021.NEBNext06.HCLNVDRXX.s_2.r_1.fq.gz
SLX-18021.NEBNext06.HCLNVDRXX.s_2.r_2.fq.gz
SLX-18021.NEBNext07.H7TY5DRXX.s_2.r_1.fq.gz
SLX-18021.NEBNext07.H7TY5DRXX.s_2.r_2.fq.gz
SLX-18021.NEBNext07.HCLNVDRXX.s_2.r_1.fq.gz
SLX-18021.NEBNext07.HCLNVDRXX.s_2.r_2.fq.gz
SLX-18021.NEBNext08.H7TY5DRXX.s_2.r_1.fq.gz
SLX-18021.NEBNext08.H7TY5DRXX.s_2.r_2.fq.gz
SLX-18021.NEBNext08.HCLNVDRXX.s_2.r_1.fq.gz
SLX-18021.NEBNext08.HCLNVDRXX.s_2.r_2.fq.gz
SLX-18021.NEBNext09.H7TY5DRXX.s_2.r_1.fq.gz
SLX-18021.NEBNext09.H7TY5DRXX.s_2.r_2.fq.gz
SLX-18021.NEBNext09.HCLNVDRXX.s_2.r_1.fq.gz
SLX-18021.NEBNext09.HCLNVDRXX.s_2.r_2.fq.gz
SLX-18021.NEBNext10.H7TY5DRXX.s_2.r_1.fq.gz
SLX-18021.NEBNext10.H7TY5DRXX.s_2.r_2.fq.gz
SLX-18021.NEBNext10.HCLNVDRXX.s_2.r_1.fq.gz
SLX-18021.NEBNext10.HCLNVDRXX.s_2.r_2.fq.gz
SLX-18021.NEBNext11.H7TY5DRXX.s_2.r_1.fq.gz
SLX-18021.NEBNext11.H7TY5DRXX.s_2.r_2.fq.gz
SLX-18021.NEBNext11.HCLNVDRXX.s_2.r_1.fq.gz
SLX-18021.NEBNext11.HCLNVDRXX.s_2.r_2.fq.gz
SLX-18021.NEBNext12.H7TY5DRXX.s_2.r_1.fq.gz
SLX-18021.NEBNext12.H7TY5DRXX.s_2.r_2.fq.gz
SLX-18021.NEBNext12.HCLNVDRXX.s_2.r_1.fq.gz
SLX-18021.NEBNext12.HCLNVDRXX.s_2.r_2.fq.gz
SLX-18021.NEBNext13.H7TY5DRXX.s_2.r_1.fq.gz
SLX-18021.NEBNext13.H7TY5DRXX.s_2.r_2.fq.gz
SLX-18021.NEBNext13.HCLNVDRXX.s_2.r_1.fq.gz
SLX-18021.NEBNext13.HCLNVDRXX.s_2.r_2.fq.gz
SLX-18021.NEBNext14.H7TY5DRXX.s_2.r_1.fq.gz
SLX-18021.NEBNext14.H7TY5DRXX.s_2.r_2.fq.gz
SLX-18021.NEBNext14.HCLNVDRXX.s_2.r_1.fq.gz
SLX-18021.NEBNext14.HCLNVDRXX.s_2.r_2.fq.gz
SLX-18021.NEBNext15.H7TY5DRXX.s_2.r_1.fq.gz
SLX-18021.NEBNext15.H7TY5DRXX.s_2.r_2.fq.gz
SLX-18021.NEBNext15.HCLNVDRXX.s_2.r_1.fq.gz
SLX-18021.NEBNext15.HCLNVDRXX.s_2.r_2.fq.gz
SLX-18021.NEBNext16.H7TY5DRXX.s_2.r_1.fq.gz
SLX-18021.NEBNext16.H7TY5DRXX.s_2.r_2.fq.gz
SLX-18021.NEBNext16.HCLNVDRXX.s_2.r_1.fq.gz
SLX-18021.NEBNext16.HCLNVDRXX.s_2.r_2.fq.gz
SLX-18021.NEBNext18.H7TY5DRXX.s_2.r_1.fq.gz
SLX-18021.NEBNext18.H7TY5DRXX.s_2.r_2.fq.gz
SLX-18021.NEBNext18.HCLNVDRXX.s_2.r_1.fq.gz
SLX-18021.NEBNext18.HCLNVDRXX.s_2.r_2.fq.gz
SLX-18021.NEBNext19.H7TY5DRXX.s_2.r_1.fq.gz
SLX-18021.NEBNext19.H7TY5DRXX.s_2.r_2.fq.gz
SLX-18021.NEBNext19.HCLNVDRXX.s_2.r_1.fq.gz
SLX-18021.NEBNext19.HCLNVDRXX.s_2.r_2.fq.gz
SLX-18021.NEBNext20.H7TY5DRXX.s_2.r_1.fq.gz
SLX-18021.NEBNext20.H7TY5DRXX.s_2.r_2.fq.gz
SLX-18021.NEBNext20.HCLNVDRXX.s_2.r_1.fq.gz
SLX-18021.NEBNext20.HCLNVDRXX.s_2.r_2.fq.gz
SLX-18021.NEBNext21.H7TY5DRXX.s_2.r_1.fq.gz
SLX-18021.NEBNext21.H7TY5DRXX.s_2.r_2.fq.gz
SLX-18021.NEBNext21.HCLNVDRXX.s_2.r_1.fq.gz
SLX-18021.NEBNext21.HCLNVDRXX.s_2.r_2.fq.gz
SLX-18021.NEBNext22.H7TY5DRXX.s_2.r_1.fq.gz
SLX-18021.NEBNext22.H7TY5DRXX.s_2.r_2.fq.gz

SLX-18021.NEBNext22.HCLNVDRXX.s_2.r_1.fq.gz
SLX-18021.NEBNext22.HCLNVDRXX.s_2.r_2.fq.gz
SLX-18021.NEBNext23.H7TY5DRXX.s_2.r_1.fq.gz
SLX-18021.NEBNext23.H7TY5DRXX.s_2.r_2.fq.gz
SLX-18021.NEBNext23.HCLNVDRXX.s_2.r_1.fq.gz
SLX-18021.NEBNext23.HCLNVDRXX.s_2.r_2.fq.gz
SLX-18021.NEBNext25.H7TY5DRXX.s_2.r_1.fq.gz
SLX-18021.NEBNext25.H7TY5DRXX.s_2.r_2.fq.gz
SLX-18021.NEBNext25.HCLNVDRXX.s_2.r_1.fq.gz
SLX-18021.NEBNext25.HCLNVDRXX.s_2.r_2.fq.gz
SLX-18021.NEBNext27.H7TY5DRXX.s_2.r_1.fq.gz
SLX-18021.NEBNext27.H7TY5DRXX.s_2.r_2.fq.gz
SLX-18021.NEBNext27.HCLNVDRXX.s_2.r_1.fq.gz
SLX-18021.NEBNext27.HCLNVDRXX.s_2.r_2.fq.gz
SLX-17459.NEBNext22.HCGCMDRXX.s_1.r_1.fq.gz
SLX-17459.NEBNext22.HCGCMDRXX.s_1.r_2.fq.gz
SLX-17459.NEBNext23.HCGCMDRXX.s_1.r_1.fq.gz
SLX-17459.NEBNext23.HCGCMDRXX.s_1.r_2.fq.gz
CTCF_Growing_1.bw
CTCF_Growing_2.bw
CTCF_Growing_3.bw
CTCF_RIS_1.bw
CTCF_RIS_2.bw
CTCF_RIS_3.bw
CTCF_Growing_4.bw
CTCF_RIS_4.bw
CTCF_RIS_DRB_1.bw
CTCF_RIS_DRB_2.bw
CTCF_TNFa_1.bw
CTCF_TNFa_2.bw
H3K27me3_Growing.bw
H3K27me3_RIS.bw
RAD21_Growing_1.bw
RAD21_Growing_2.bw
RAD21_Growing_3.bw
RAD21_RIS_1.bw
RAD21_RIS_2.bw
RAD21_RIS_3.bw
RAD21_RIS_4.bw
RAD21_RIS_5.bw
RAD21_RIS_DRB_1.bw
RAD21_RIS_DRB_2.bw
SMC3_Growing_1.bw
SMC3_Growing_2.bw
SMC3_pBabe_RAS.bw
SMC3_pBabe_v.bw
SMC3_RIS_1.bw
SMC3_RIS_2.bw
SMC3_RIS_3.bw
SMC3_RIS_DRB_1.bw
SMC3_RIS_DRB_2.bw
WI38_SMC3_pBabe_RAS.bw
WI38_SMC3_pBabe_v.bw
RAD21_Growing_4.bw
RAD21_TNFa_1.bw
RAD21_TNFa_2.bw
SMC3_Growing_3.bw
SMC3_TNFa_1.bw
SMC3_TNFa_2.bw
H3K27ac_Growing.bw
H3K27ac_TNFa.bw
PolII_Growing_1_l1.fq.gz
PolII_Growing_1_l2.fq.gz
PolII_Growing_2_l1.fq.gz
PolII_Growing_2_l2.fq.gz
PolII_Growing_3_l1.fq.gz
PolII_Growing_3_l2.fq.gz
PolII_Growing_4_l1.fq.gz

PolII_Growing_4_l2.fq.gz
PolII_RIS_1_l1.fq.gz
PolII_RIS_1_l2.fq.gz
PolII_RIS_2_l1.fq.gz
PolII_RIS_2_l2.fq.gz
PolII_RIS_3_l1.fq.gz
PolII_RIS_3_l2.fq.gz
PolII_RIS_4_l1.fq.gz
PolII_RIS_4_l2.fq.gz
Input_Growing_1_l1.fq.gz
Input_Growing_1_l2.fq.gz
Input_Growing_2_l1.fq.gz
Input_Growing_2_l2.fq.gz
Input_Growing_3_l1.fq.gz
Input_Growing_3_l2.fq.gz
Input_RIS_1_l1.fq.gz
Input_RIS_1_l2.fq.gz
Input_RIS_2_l1.fq.gz
Input_RIS_2_l2.fq.gz
Input_RIS_3_l1.fq.gz
Input_RIS_3_l2.fq.gz
PolII_Growing_1.bw
PolII_RIS_1.bw
PolII_Growing_2.bw
PolII_RIS_2.bw
PolII_Growing_3.bw
PolII_RIS_3.bw
PolII_Growing_4.bw
PolII_RIS_4.bw

Genome browser session
(e.g. UCSC)

Tracks with normalized ChIP-seq signal (THOR: library normalized and input subtracted) were submitted to GEO which can be downloaded and visualized with the genome browser of choice.

Methodology

Replicates

CTCF ChIP-seq consisted of four replicates for growing (control) IMR90 cells and RAS-induced Senescence (RIS) IMR90 cells, respectively, two replicates of CTCF in TNFa treated IMR90 cells and two replicates of CTCF in RIS IMR90 cells with DRB treatment.
Cohesin ChIP-seq consisted of four replicates of RAD21 in growing IMR90 cells, five replicates of RAD21 in RIS IMR90 cells, three replicates of SMC3 in growing IMR90 cells, three replicates of SMC3 in RIS IMR90 cells, two replicates of RAD21 in growing IMR90 cells with TNFa treatment, two replicates of SMC3 in growing with TNFa treatment IMR90 cells, two replicates of RAD21 in RIS IMR90 cells with DRB treatment, two replicates of SMC3 in RIS IMR90 cells with DRB treatment, one replicate of SMC3 in growing IMR90 cells with empty vector and one SMC3 replicate in RIS (pBabe induced) IMR90 cells, one replicate of SMC3 in growing WI38 cells with empty vector and one SMC3 replicate in RIS (pBabe induced) WI38 cells.
ChIP-seq for H3K27me3 consisted of one growing and one RIS replicates and was matched with ChIP-seq data from a previous study (Chandra et al. 2012, mentioned in Methods) performed using the same system (RAS-induced Senescence against control growing cells). ChIP-seq for H3K27ac consisted of one replicate corresponding to growing cells and one corresponding to cells treated with TNFa. ChIP-seq for Pol II consisted of 4 growing and 4 RIS replicates.

Replicates agree with over 0.7 Pearson correlation, were checked for clustering between conditions with ChIPQC (R Bioconductor and a large number of peaks coincide between each pair of replicates in each case, calculated using a consensus peak set for each condition, defined as the set of peaks which appears in at least two replicates: growing CTCF - consensus set consists of 44,764 peaks, CTCF RIS - 53,563 peaks, cohesin RAD21 growing - 26,374, cohesin RAD21 RIS - 24,355).

Sequencing depth

All experiments involved reads of length 50; SLX-12520, SLX-15808 and SLX-15809 were single-end and SLX-18021 and SLX-17459 were paired-end.
Number of uniquely aligned reads for each condition:
CTCF (Growing): 36622966, 28481947, 33678492, 70059087
CTCF (RIS): 30059687, 29453455, 24921054, 68382611
CTCF (TNFa treatment): 64658657, 71766990
CTCF (RIS + DRB treatment): 82859607, 71922128
Cohesin (RAD21 Growing): 14828639, 14021002, 78736472, 68367908
Cohesin (RAD21 RIS): 60764643, 71558651, 96829246, 91280349, 82977231
Cohesin (SMC3 Growing): 15038851, 113541102, 66107504
Cohesin (SMC3 RIS): 69266728, 19429200, 70856937
Cohesin (RAD21 TNFa treatment): 76546983, 56675747
Cohesin (SMC3 TNFa treatment): 69161876, 72183753
Cohesin (RAD21 RIS + DRB treatment): 67842130, 63769474
Cohesin (SMC3 RIS + DRB treatment): 48188995,65494193

	Cohesin (SMC3 WI38 empty vector): 65306179<br>Cohesin (SMC3 WI38 pBabeRAS): 64070615<br>Cohesin (SMC3 IMR90 empty vector): 68306333<br>Cohesin (SMC3 IMR90 pBabeRAS): 66188745<br>H3K27ac (Growing): 72578775<br>H3K27ac (TNFa treatment): 42102471<br>H3K27me3 (Growing): 85612821<br>H3K27me3 (RIS): 65618362
Antibodies	anti-H3K27ac (Clone CMA309)<br>anti-H3K27me3 (Clone CMA323)<br>anti-CTCF (clone D31H12, Cell Signaling Technology, #3418)<br>anti-RAD21 (gift from Shirahige lab)<br>anti-SMC3 (Abcam, ab9263)<br>anti-PolII (Clone C13B9)
Peak calling parameters	macs2 callpeak -t **ChIPsample.bam** -c **matchedInput** -f BAM -g hs --nomodel --extsize **as determined with ChIPQC** --outdir Peaks/ -n **SampleName**<br>In the case of H3K27me3 we used the option --broad.
Data quality	Quality of the data was checked using the ChIPQC R Bioconductor package, replicates from the same condition correlated well (above 0.75 Pearson correlation). We generated consensus peak sets defined as peaks which appear in at least two replicates and the number of peaks common between each replicate of control samples was comparable to number of CTCF/cohesin peaks reported in other studies.
Software	We aligned ChIP-seq data using bowtie2, converted the alignments from SAM to BAM format using samtools, removed duplicates and filtered blacklisted regions using samtools and
